# Prediction of the risk of mortality in older patients with coronavirus disease 2019 using blood markers and machine learning

**DOI:** 10.3389/fimmu.2024.1445618

**Published:** 2024-11-01

**Authors:** Linchao Zhu, Yimin Yao

**Affiliations:** Medical Laboratory, The First Affiliated Hospital of Zhejiang Chinese Medical University (Zhejiang Provincial Hospital of Chinese Medicine), Hangzhou, China

**Keywords:** COVID-19, older patients, mortality, blood markers, machine learning, Gaussian naïve Bayes

## Abstract

**Introduction:**

The mortality rate among older people infected with severe acute respiratory syndrome coronavirus 2 is alarmingly high. This study aimed to explore the predictive value of a novel model for assessing the risk of death in this vulnerable cohort.

**Methods:**

We enrolled 199 older patients with coronavirus disease 2019 (COVID-19) from Zhejiang Provincial Hospital of Chinese Medicine (Hubin) between 16 December 2022 and 17 January 2023. Additionally, 90 patients from two other centers (Qiantang and Xixi) formed an external independent testing cohort. Univariate and multivariate analyses were used to identify the risk factors for mortality. Least absolute shrinkage and selection operator (LASSO) regression analysis was used to select variables associated with COVID-19 mortality. Nine machine-learning algorithms were used to predict mortality risk in older patients, and their performance was assessed using receiver operating characteristic curves, area under the curve (AUC), calibration curve analysis, and decision curve analysis.

**Results:**

Neutrophil–monocyte ratio, neutrophil–lymphocyte ratio, C‐ reactive protein, interleukin 6, and D-dimer were considered to be relevant factors associated with the death risk of COVID-19-related death by LASSO regression. The Gaussian naive Bayes model was the best-performing model. In the validation cohort, the model had an AUC of 0.901, whereas in the testing cohort, the model had an AUC of 0.952. The calibration curve showed a good correlation between the actual and predicted probabilities, and the decision curve indicated a strong clinical benefit. Furthermore, the model had an AUC of 0.873 in an external independent testing cohort.

**Discussion:**

In this study, a predictive machine-learning model was developed with an online prediction tool designed to assist clinicians in evaluating mortality risk factors and devising targeted and effective treatments for older patients with COVID-19, potentially reducing the mortality rates.

## 1 Introduction

Severe acute respiratory syndrome coronavirus-2 (SARS-CoV-2) has posed a significant global health challenge since December 2019 with the coronavirus disease 2019 (COVID-19) pandemic spreading rapidly worldwide. As of 12 February 2024, the infection has affected over 760 million individuals worldwide, resulting in 6.9 million deaths (1). COVID-19 manifests as a spectrum of clinical symptoms, from asymptomatic or mild cases to severe presentations such as pneumonia, respiratory distress syndrome, or death (2). Notably, older adults experience higher severity and mortality rates than younger people, with those over the age of 85 years facing a 330-fold higher risk of death (3). Immunosenescence and the presence of underlying chronic diseases contribute to the heightened vulnerability of older people to severe COVID-19 outcomes (4–6). It is imperative to establish robust laboratory diagnostics to mitigate the risk of mortality among older patients.

Machine learning (ML) is of great value in the medical field, and many studies have utilized machine learning as a tool to identify and classify data, aiming to identify individual features of data from ML, establish models through science, and subsequently utilize new data through these models to forecast future data (7). Machine-learning algorithms have been employed as an effective research approach for predicting the spread of deadly infectious diseases, such as COVID-19 (8). Moulaei et al. (9) found that ML-based predictive models, particularly the random forest (RF) algorithm, potentially facilitate the identification of patients who are at high risk of mortality and inform appropriate interventions by clinicians using the most important clinical features (dyspnea, intensive care unit admission, and oxygen therapy). However, there is a lack of predictive models based on data from basic laboratory blood tests that focus on mortality. Predictive models can identify patients who are at increased risk of mortality and provide support to reduce deaths as soon as possible, which can provide more accurate predictions than relying on single factors or doctors’ intuitive judgments. Many studies have shown that inflammation and a hypercoagulable state play a significant role in older patients who are more prone to death from COVID-19 (10, 11). Neutrophil (NEU) count, lymphocyte (LYM) count, monocyte (MON) count, neutrophil–lymphocyte ratio (NLR), neutrophil–monocyte ratio (NMR), lymphocyte–monocyte ratio (LMR), C‐reactive protein (CRP) level, procalcitonin (PCT) level, and interleukin 6 (IL6) level are common indicators of systemic inflammation, and D-dimer (DD) is a hypercoagulable marker as well as an indicator for monitoring inflammation and severe infection (12–14). Laboratory blood tests are more convenient, economical, and less invasive than medical imaging, and the above indicators are obtained from basic and routine clinical blood tests that can be acquired at short notice.

In this study, we collected and analyzed inflammation and hypercoagulable indicators to build a predictive ML model that can assist clinicians in predicting the risk factors associated with mortality and in developing unique and effective treatment approaches for older patients infected with SARS-CoV-2.

## 2 Materials and methods

### 2.1 General information

This study was approved by the Ethics Committee of The First Affiliated Hospital of Zhejiang Chinese Medical University (reference number 2023-KLS-034-01). We conducted a retrospective analysis of 199 patients with COVID-19 diagnosed and treated at the Zhejiang Provincial Hospital of Chinese Medicine (Hubin) and 90 patients from two additional centers (Qiantang and Xixi) between 16 December 2022 and 17 January 2023. Patient data, including electronic medical records and laboratory indices, were obtained from the hospital information system. The inclusion criteria were as follows: 1) availability of complete clinical data, 2) age of 60 years or older, and 3) a positive real-time polymerase chain reaction result for SARS-CoV-2 RNA. On the other hand, the exclusion criteria were as follows: 1) presence of other infections, 2) co-occurrence with malignant tumors or severe blood/immune system disorders, and 3) receipt of blood transfusion within the previous month.

### 2.2 Data collection

Detailed clinical information and data of the subjects were collected, including age, sex, comorbidities, routine peripheral blood examination (NEU count, LYM count, and MON count), CRP, DD, PCT, and IL6. The NLR, NMR, and LMR values were calculated as follows: NLR = NEU  count  (10^9^/L)/LYM  count  (10^9^/L); NMR = NEU  count  (10^9^/L)/MON count  (10^9^/L); LMR = LYM  count   (10^9^/L)/MON count  (10^9^/L). Peripheral blood was collected upon admission and centrifuged at 3,000*g*/min for 5 min to detect the above markers, and the results were available within 2 h. Based on patient outcomes, the 199 patients were divided into two groups: discharged group and deceased group. After enrollment, the 199 individuals were randomly assigned to the training cohort (75%, including 15% of the validation cohort) and the testing cohort (25%) for ML. By setting a random seed (random seed = 1), repeatability of the random process was ensured, allowing us to accurately reproduce the results when required. The random forest filling method was used to fill the data of age, NEU count, LYM count, MON count, NLR, NMR, LMR, CRP, PCT, IL6, and DD before training. The best model hyperparameters were selected by a grid search and a 10-fold cross-validation was performed. Ninety patients from the two other centers (Qiantang and Xixi) were included as an external independent testing cohort.

### 2.3 Machine learning

The Beckman Coulter DxAI platform (https://www.xsmartanalysis.com/beckman/login/) was used for statistical analysis. Least absolute shrinkage and selection operator (LASSO) regression analysis was used to identify factors associated with COVID-19 mortality. The optimal ML model was selected from nine candidates: XGBoost, logistic regression (LR), LightGBM (LGBM), RF, AdaBoost, decision tree (DT), gradient boosting decision tree (GBDT), Gaussian naive Bayes (GNB), and complement naive Bayes (CNB). The models were evaluated based on calibration plots, and their predictive performance was assessed using sensitivity, specificity, accuracy, predictive value, and the area under the curve (AUC) in both the testing and validation cohorts. The filtered model was validated using an external independent testing cohort.

### 2.4 Statistical analysis

Statistical analyses were performed using SPSS 16.0 and R version V4.2.3. Student’s *t*-test and the Wilcoxon signed-rank test were used to analyze measurement data, and the chi-square test was used to analyze count data between two groups. Numerical variables with normal distribution and homogeneity of variance were compared using Student’s *t*-test, whereas numerical variables with a normal distribution and heterogeneity of variance were compared using the Wilcoxon signed-rank test. LASSO regression analysis was performed to identify the factors associated with COVID-19-related death. The performance of these factors was assessed using receiver operating characteristic (ROC) curves. Statistical significance was set at *P <*0.05.

## 3 Results

### 3.1 Baseline clinical characteristics

The baseline characteristics of the 199 patients are shown in **Table 1**. Of these, 157 (78.89%) were categorized as having recovered and were discharged, including 89 male patients (56.69%). There were 42 (21.11%) patients in the deceased group, including 30 male patients (71.43%). No significant sex differences for all laboratory indices were observed (*P* = 0.084). The mean age of the patients in the deceased group was significantly higher than that of the patients in the discharged group (*P* = 0.002). Hypertension was the most prevalent comorbidity in the deceased group, followed by diabetes, coronary heart disease, and renal dysfunction. Compared with the discharged group, the deceased group had significantly higher NEU counts, NLR, NMR, CRP, PCT, IL6, and DD. There were also markedly lower LYM and MON counts and lower LMR (*P* < 0.05).

**Table 1:** The baseline clinical feature of COVID-19 patients.

Characteristics	Discharged group (*n* = 157)	Deceased group (*n* = 42)	*P*-value
Age, median (IQR)	81 (69, 88)	86 (79, 91)	0.002
Sex, *n* (%)			0.084
Male	89 (56.69%)	30 (71.43%)	
Female	68 (43.31%)	12 (28.57%)	
Comorbidities, *n* (%)			
Diabetes	16 (10.19%)	5 (11.90%)	
Hypertension	95 (60.51%)	24 (57.14%)	
Coronary heart disease	8 (5.09%)	2 (4.76%)	
Renal dysfunction	5 (3.18%)	1 (2.38%)	
Others	33 (21.02%)	10 (23.81%)	
Variable category			
Neutrophil (10^9^/L), median (IQR)	4.90 (3.2, 6.2)	6.80 (5.0, 11.8)	<0.001
Lymphocyte (10^9^/L), median (IQR)	0.80 (0.6, 1.0)	0.50 (0.4, 0.7)	<0.001
Monocytes (10^9^/L), median (IQR)	0.50 (0.4, 0.6)	0.40 (0.3, 0.5)	0.007
NLR, median (IQR)	6.10 (3.36, 8.50)	15.00 (9.88, 22.50)	<0.001
NMR, median (IQR)	9.50 (6.50, 13.75)	21.50 (15.75, 28.00)	<0.001
LMR, median (IQR)	1.67 (1.25, 2.25)	1.50 (1.00, 1.75)	0.017
CRP (mg/L), median (IQR)	37.50 (15.57, 60.48)	109.56 (70.55, 149.67)	<0.001
PCT (μg/L), median (IQR)	0.22 (0.15, 0.38)	0.89 (0.28, 1.72)	<0.001
IL6 (pg/L), median (IQR)	18.45 (11.98, 30.84)	37.22 (22.45, 96.56)	<0.001
DD, (mg/L), median (IQR)	1.11 (0.62, 1.63)	2.02 (1.56, 6.05)	<0.001

### 3.2 Feature selection correlated with COVID-19-related death

**Figure 1:**
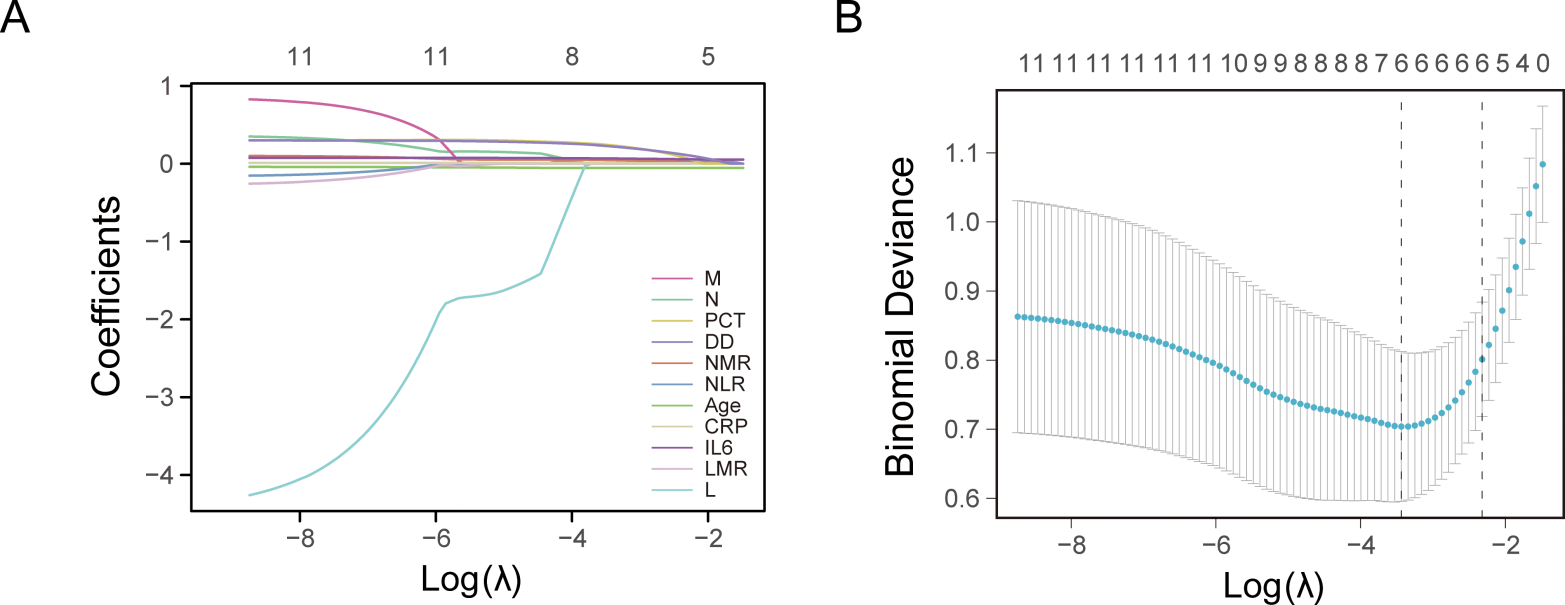
Least absolute shrinkage and selection operator (LASSO) regression analysis and 10-fold cross-validation for selecting factors associated with the death of coronavirus disease 2019 (COVID-19) elderly patients. **(A)** Bias selection of the tuning parameter (lambda) in LASSO regression based on the minimum standard (left dashed line) and 1-SE (standard error) standard (right dashed line). **(B)** A joint plot was created based on the log-likelihood.

**Figure 2:**
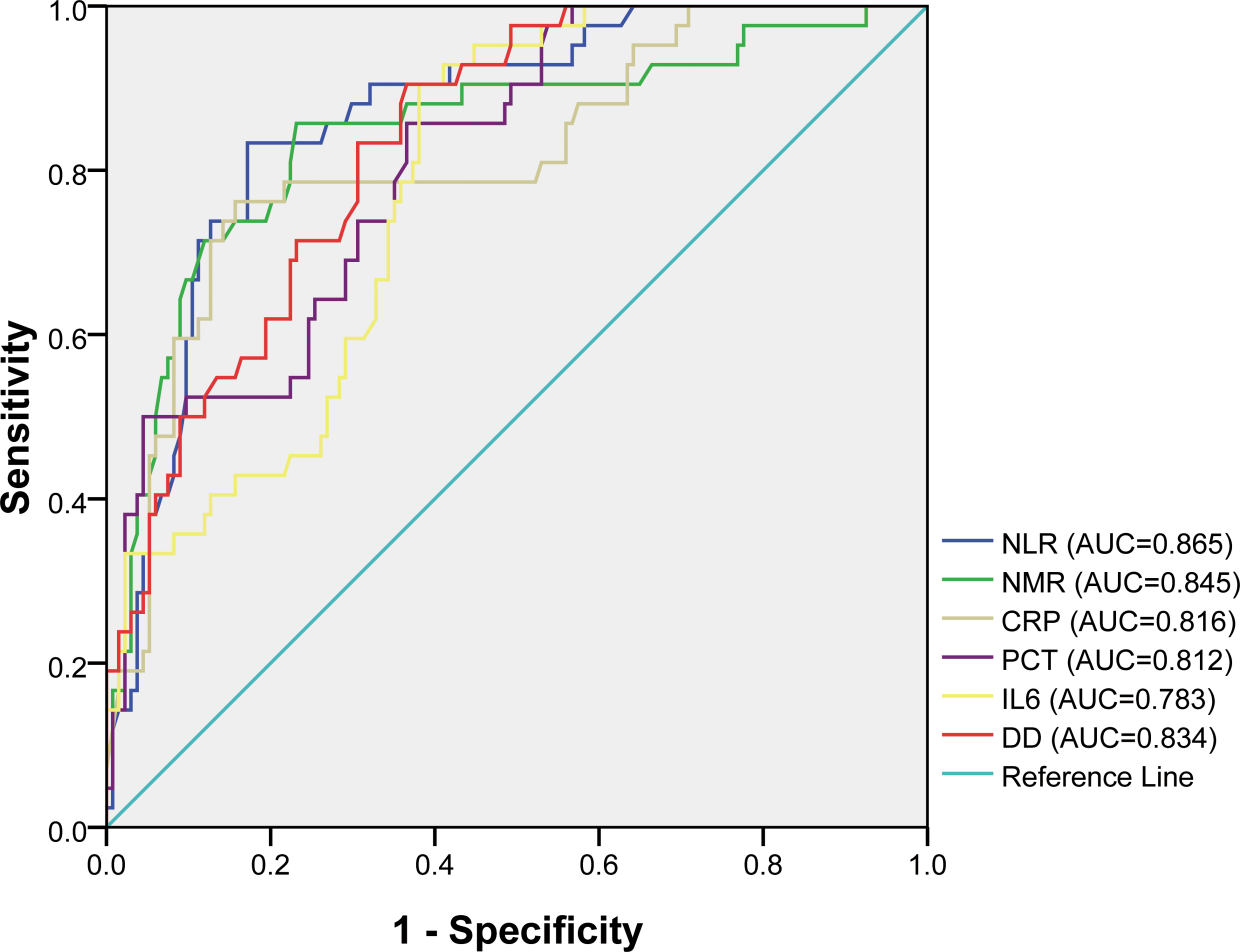
Receiver operating characteristic (ROC) curves for different factors in predicting the risk of mortality of COVID-19 elderly patients.

LASSO regression analysis was conducted to identify factors influencing COVID-19 mortality (**Table 1**). NMR, NLR, CRP, IL6, PCT, and DD were identified as relevant factors associated with the risk of COVID-19-related death (**Figure 1**). Furthermore, we analyzed the AUC of these factors using ROC analysis (**Figure 2**) to predict the risk of mortality in older individuals with COVID-19 between the two groups. The AUC values for the NMR, NLR, CRP, IL6, PCT, and DD were 0.845, 0.865, 0.816, 0.783, 0.812, and 0.834, respectively.

### 3.3 Features identified using ML algorithms

**Figure 3:**
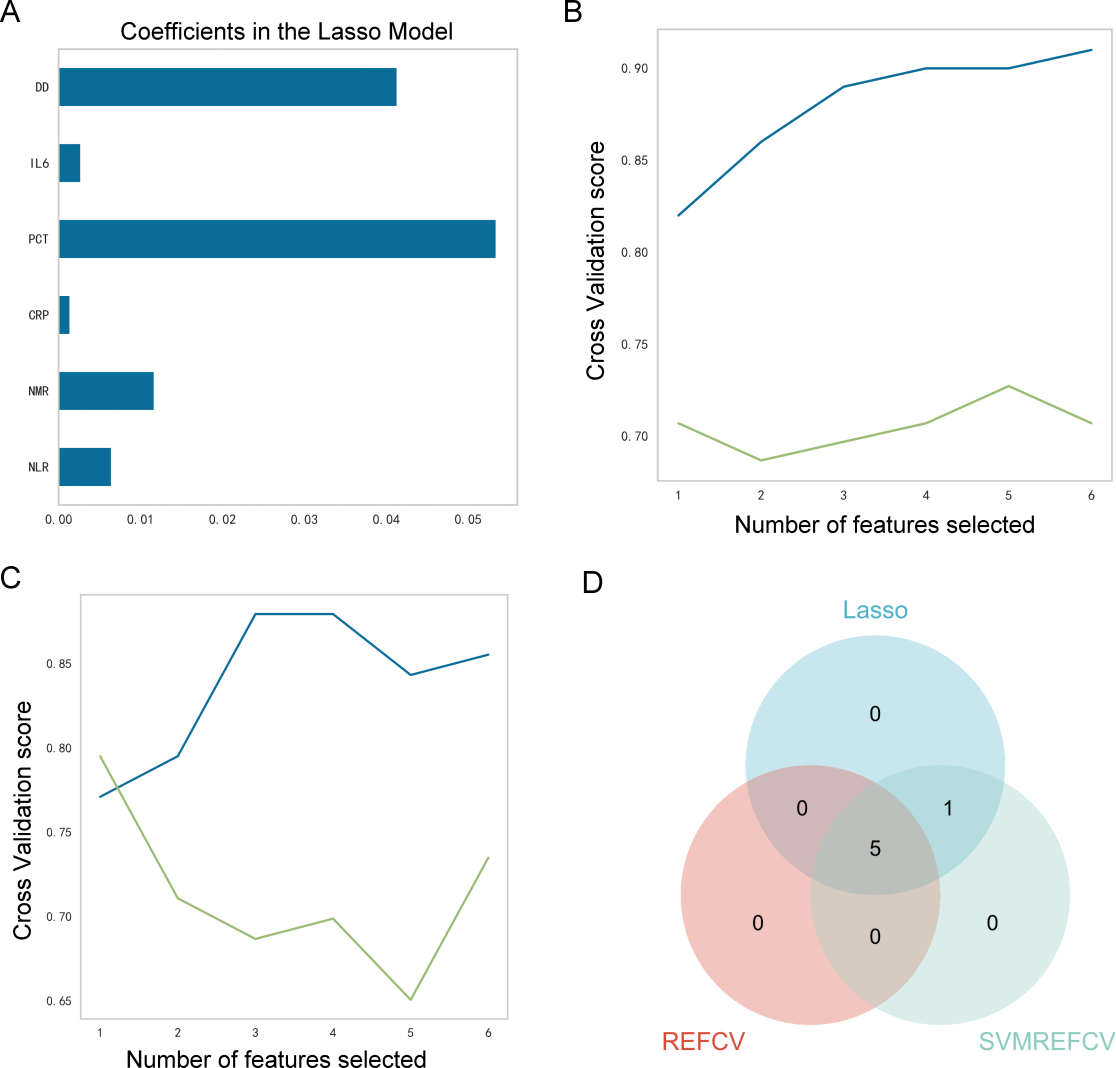
Identification of characteristic markers. **(A)** Six markers were identified using the LASSOCV algorithm; **(B)** five markers were identified using the REFCV algorithm; **(C)** six markers were identified using the SVMREFCV algorithm; **(D)** Venn plot of markers for three machine-learning algorithms.

The LASSOCV, REFCV, and SVMREFCV algorithms were used to identify markers (**Figures 3A–C**, respectively), and Venn diagrams were drawn in the R language (**Figure 3D**). After considering the intersection of the three algorithms, five overlapping markers were identified: NMR, NLR, CRP, IL6, and DD.

### 3.4 Identification of the optimal ML model

**Figure 4:**
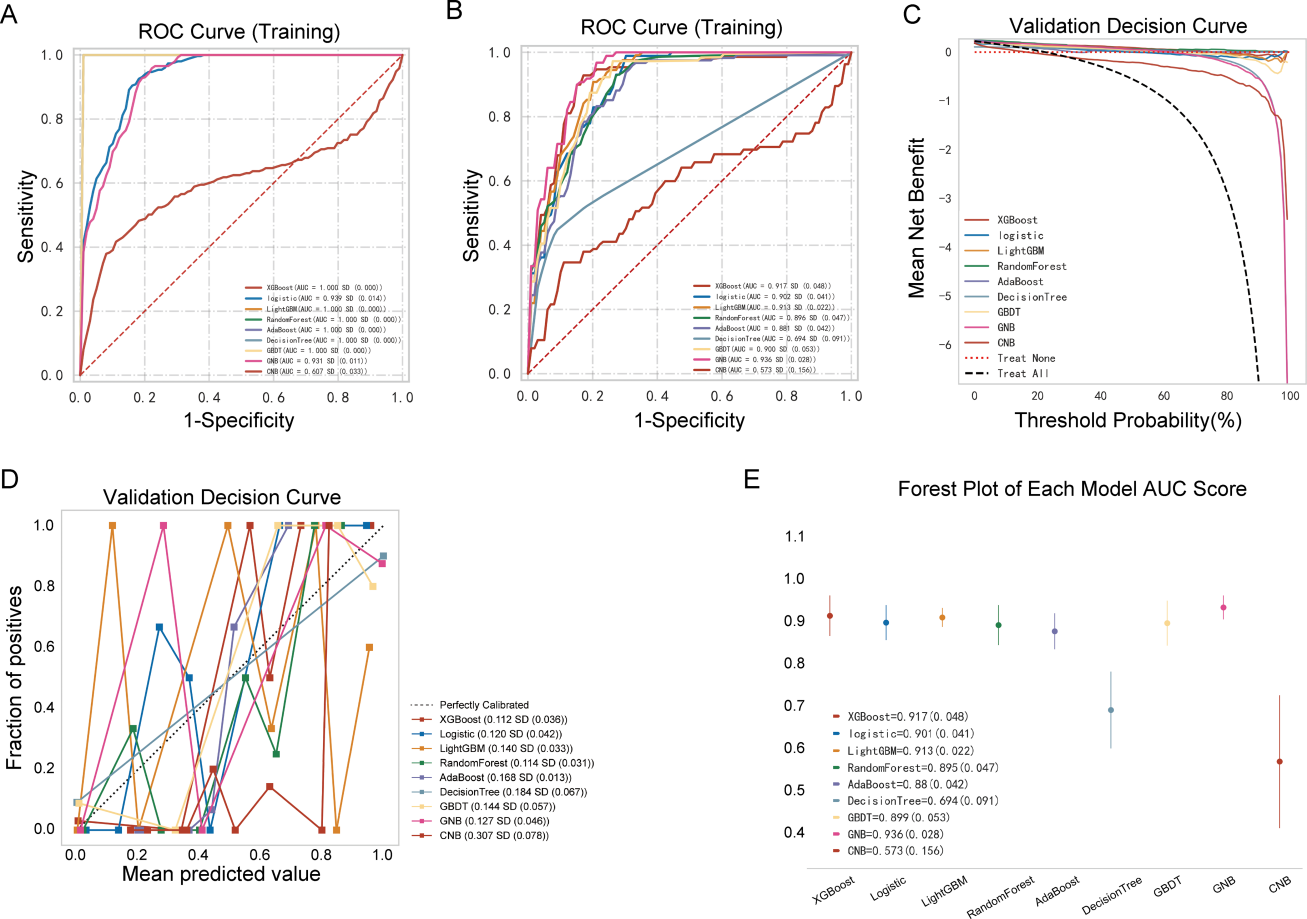
Performance comparison between multiple models. **(A)** Receiver operating characteristic (ROC) curve of the training cohort; **(B)** ROC curve of the validation cohort; **(C)** decision curve of the nine machine-learning models; **(D)** calibration curve of the nine machine-learning models; **(E)** forest plot of each area under the curve (AUC) score.

The performances of the nine ML models in the training and validation cohorts are presented in **Table 2**, **Figure 4**. The GNB model demonstrated the highest predictive accuracy with AUC values of 0.924 and 0.936 in the validation and testing cohorts, respectively. Calibration and decision curves confirmed the superior performance and clinical utility of the GNB model.

**Table 2:** Diagnostic efficacy of nine classifiers in the training and validation cohorts.

Classifier	Cohorts	AUC	Cutoff	Accuracy	Sensitivity	Specificity	Positive predictive value	Negative predictive value	F1
XGBoost	Training	1.000	0.759	0.992	1.000	1.000	1.000	0.990	1.000
	Validation	0.917	0.759	0.819	0.941	0.869	0.749	0.828	0.806
Logistic	Training	0.939	0.182	0.852	0.953	0.829	0.638	0.971	0.761
	Validation	0.902	0.182	0.795	0.989	0.790	0.459	0.959	0.610
LightGBM	Training	1.000	0.814	0.992	1.000	1.000	1.000	0.990	1.000
	Validation	0.913	0.814	0.816	0.973	0.790	0.749	0.830	0.833
Random forest	Training	1.000	0.550	0.988	1.000	0.999	1.000	0.985	1.000
	Validation	0.896	0.550	0.819	0.931	0.783	0.734	0.845	0.803
AdaBoost	Training	1.000	0.522	0.992	1.000	1.000	1.000	0.990	1.000
	Validation	0.881	0.522	0.788	0.959	0.786	0.598	0.835	0.727
Decision tree	Training	1.000	1.000	0.773	1.000	1.000	NaN	0.773	NaN
	Validation	0.694	1.000	0.781	0.477	0.912	NaN	0.781	NaN
GBDT	Training	1.000	0.935	0.992	1.000	1.000	1.000	0.990	1.000
	Validation	0.900	0.935	0.816	0.964	0.813	0.756	0.826	0.837
GNB	Training	0.931	0.047	0.838	0.962	0.812	0.595	0.974	0.735
	Validation	0.936	0.047	0.837	1.000	0.801	0.536	0.982	0.697
CNB	Training	0.607	0.813	0.768	0.479	0.871	0.553	0.834	0.495
	Validation	0.573	0.813	0.730	0.598	0.753	0.281	0.840	0.339

### 3.5 Analysis and assessment of the GNB ML model

**Figure 5:**
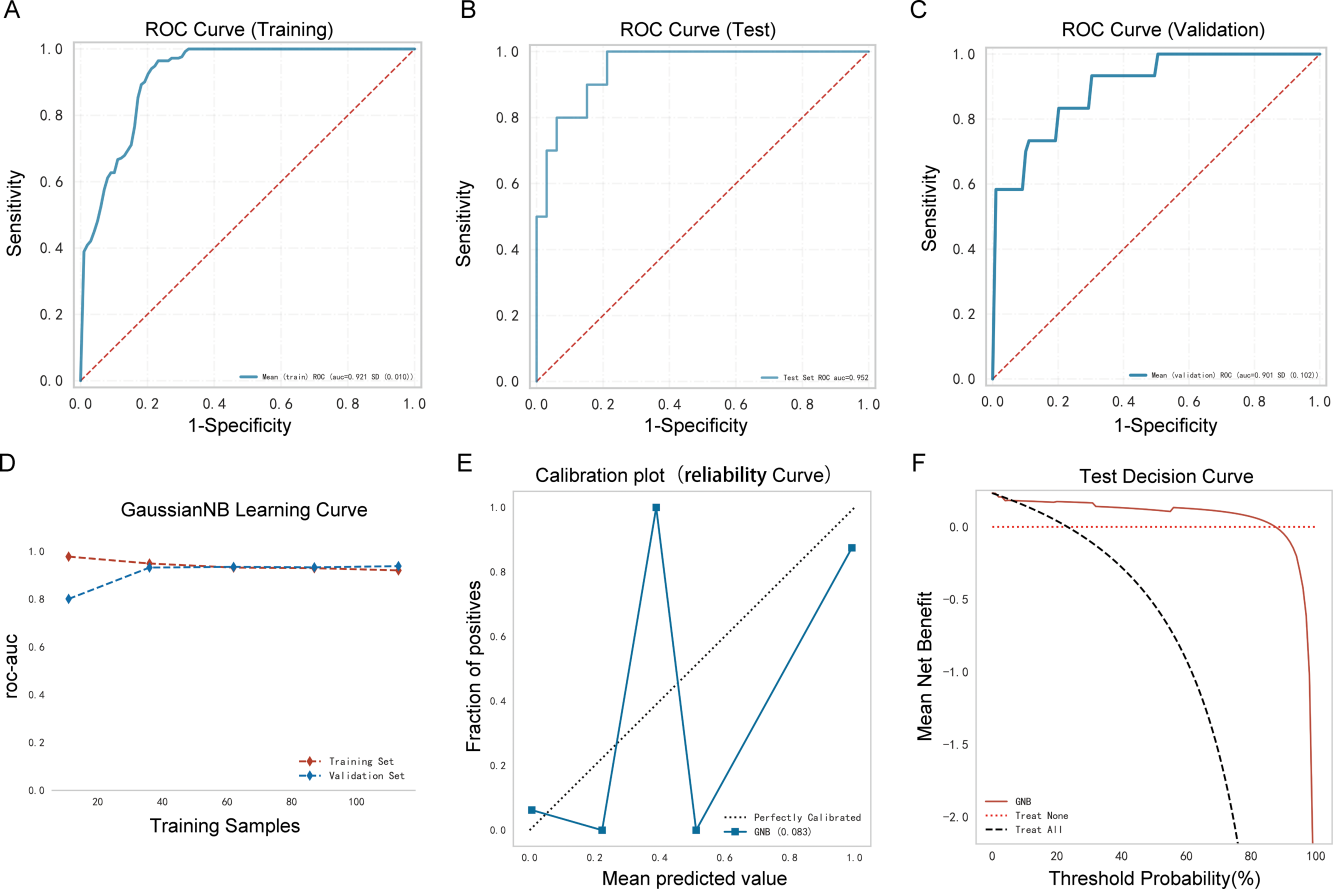
Performance of the prediction model. **(A)** Receiver operating characteristic (ROC) curve of the training cohort; **(B)** ROC curve of the validation cohort; **(C)** ROC curve of the testing cohort; **(D)** AUC of the validation cohort and the testing cohort; **(E)** calibration curve analysis; **(F)** decision curve analysis.

As shown in **Table 3**, **Figures 5A–C**, the AUC of the validation cohort did not exceed that of the testing cohort and the AUC of the validation cohort did not exceed that of the training cohort in **Figure 5D**, demonstrating that the GNB model had a strong fitting ability. **Table 3** shows that accuracy, sensitivity, and specificity exceeded 78% in the testing cohort. The differences in metrics for GNB models in **Tables 2**, **3** were due to reassignment of the data used to test the models. In addition, the calibration curve illustrates a good correlation between the actual and predicted probabilities (**Figure 5E**), and the decision curve indicates a strong clinical benefit (**Figure 5F**), indicating that GNB was an excellent model.

**Table 3:** Diagnostic efficacy of the GNB model in the testing and validation cohorts.

Cohorts	AUC	Cutoff	Accuracy	Sensitivity	Specificity	Positive predictive value	Negative predictive value	F1
Training	0.921	0.066	0.822	0.964	0.793	0.561	0.974	0.709
Validation	0.901	0.066	0.821	1.000	0.819	0.587	0.978	0.724
Testing	0.952	0.061	0.884	1.0	0.788	0.727	0.938	0.842

### 3.6 External validation of the GNB ML model

**Figure 6:**
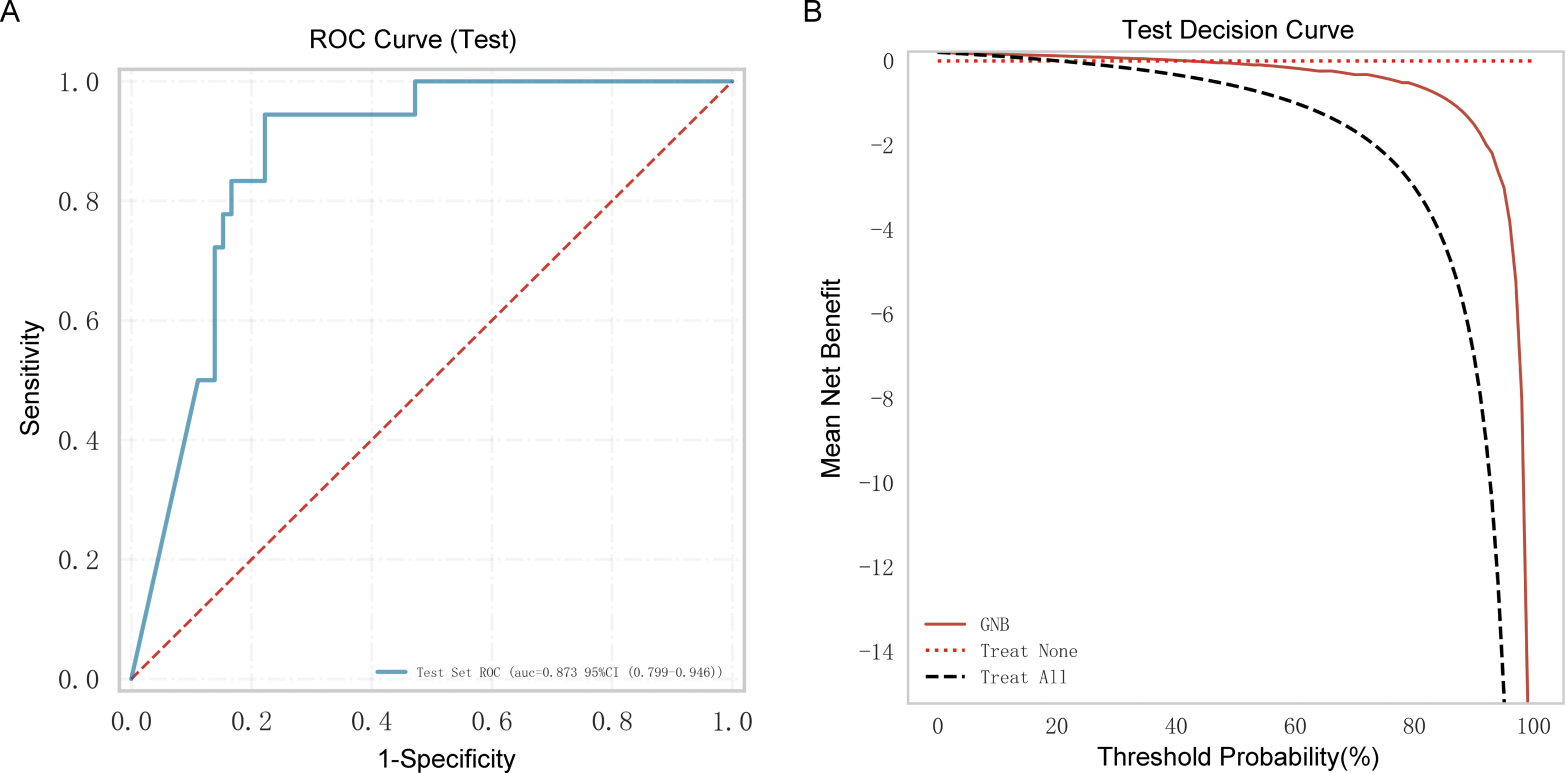
External independent testing of the Gaussian naive Bayes (GNB) regression model. **(A)** Receiver operating characteristic (ROC) curve of the external independent testing cohort. **(B)** Test decision curve of the external independent testing cohort.

Ninety older people with COVID-19 were enrolled in an external independent testing cohort from two other centers. The AUC of the model was 0.873 (**Figure 6A**), and the decision curve indicated that the tool had a strong clinical benefit (**Figure 6B**).

### 3.7 Online prediction tool

**Figure 7:**
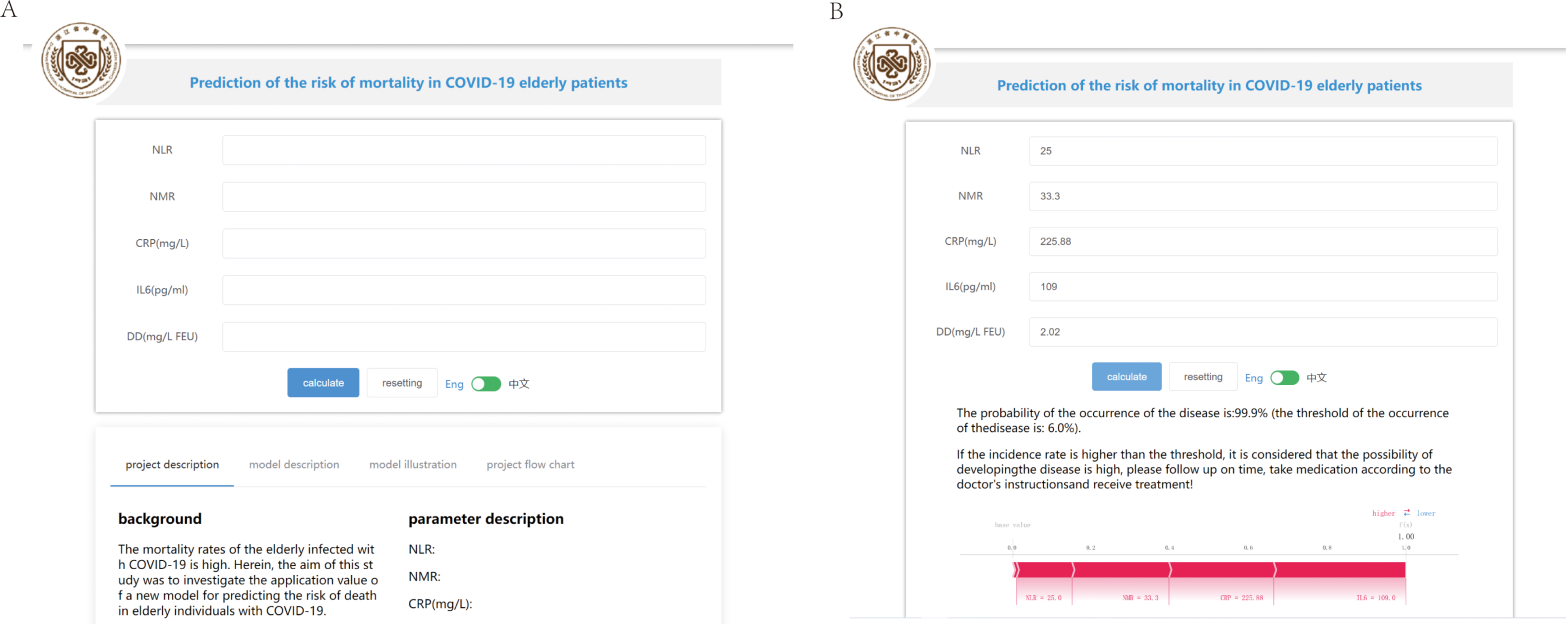
An online prediction tool to predict the risk of mortality. **(A)** An online page based on the Gaussian naive Bayes (GNB) algorithm. **(B)** An online page to predict the risk of COVID-19 mortality based on five indicators.

Based on the above analysis, an online prediction tool was built for clinicians to predict the risk of mortality in older patients with COVID-19 (http://www.xsmartanalysis.com/model/list/predict/model/html?mid=15316&symbol=41716818278ELBg2YAyL). The blood indices (NLR, NMR, CRP, IL6, and DD) were entered in the website. If the results indicate a high risk of death, clinicians should be vigilant and prepare treatment in advance (**Figure 7**).

## 4 Discussion

SARS-CoV-2 spreads very rapidly with human-to-human transmission. Although COVID-19 has a relatively low fatality rate overall, it has a higher mortality rate in older people, particularly those 80 years and older (11, 15). To date, safe and effective treatments have been lacking. Although a COVID-19 “wonder drug” is used clinically, its side effects and limitations are unavoidable; thus, it is not suitable for all patients. In recent years, there have been many studies on COVID-19, but few have focused on older people, particularly in predicting the risk of mortality in this group. Blood tests contain a large amount of information about the disease and are readily accepted by patients. To explore the potential dependencies between blood test biomarkers and COVID-19, an ML approach can be used. Currently, ML algorithms are rapidly developing as model-building tools that have been widely applied in the medical field with powerful predictive and parallel processing capabilities (16). Therefore, we developed a predictive model after assessing nine ML models (LGBM, GBDT, XGBoost, RF, GNB, AdaBoost, LR, DT, and CNB) to determine the risk of COVID-19-related death in older people using laboratory data.

This study identified five blood markers (NMR, NLR, CRP, IL6, and DD) that were significantly associated with the risk of death in patients with COVID-19. Among the nine ML algorithms tested, the GNB model showed the highest predictive accuracy, with high AUC values in both the validation and testing cohorts. The well-calibrated predictions of the model, as indicated by the calibration curve, are crucial for clinical application. Decision curve analysis further supported the clinical utility of the GNB model, indicating a high net benefit across various threshold probabilities and the ability of the model to guide clinical decision-making by accurately identifying patients who would benefit most from intensive care and targeted interventions.

SARS-CoV-2 infection induces shortness of breath more frequently in older people than in younger people (17). However, clinicians may overlook this symptom because of similar presentations due to age-related declines and comorbidities (18). Therefore, the establishment of predictive models has significant clinical utility. Our data revealed that older patients with chronic diseases were at a higher risk, with those who died having a median age of 86 years, which was significantly higher than that of the discharged patients, with a median age of 81 years (*P* < 0.01). This aligns with the findings by McMichael et al. (19) showing that hospitalization and mortality rates were higher in patients with a median age of 83 years and a 94% prevalence of underlying conditions. Notably, a higher proportion of male patients was observed in the deceased group.

Systemic inflammation plays a significant role in the progression of COVID-19 (20). Excessive inflammation may lead to a weak immune response, thereby contributing to multiple organ dysfunction. Blood indices, such as NEU count, LYM count, MON count, NLR, NMR, LMR, CRP, PCT, and IL6, can represent inflammation and immune status (21). This study showed that NLR, NMR, CRP, and IL6 in the deceased group were significantly higher than those in the discharged group (*P* < 0.05). Our study also found that NLR and NMR had good screening effects on COVID-19 prognosis and death, with an AUC of 0.865 and 0.845, respectively, which is consistent with previous reports (22, 23), indicating that these indicators were related to poor COVID-19 outcomes. CRP and IL6 are commonly used as inflammatory markers in clinical settings. In a study by Wolszczak-Biedrzycka et al. (24), the CRP level increased with disease severity, assessed based on the modified early warning score, indicating that CRP is a useful predictor of disease severity and the risk of mortality. The present study confirmed this result as the CRP level was significantly higher in patients who died from COVID-19 (*P* < 0.01), with an AUC of 0.816. According to some studies, IL6 is an excellent predictor of mortality in patients with COVID-19 (25). Excessive IL6 is secreted by CD14^+^ and CD16^+^ monocytes during COVID-19 progression, which is associated with a systemic inflammatory reaction known as a cytokine storm (26, 27). Our results align with the existing body of evidence that an elevated IL6 level is linked to an increased mortality risk (*P* < 0.01). In addition to excessive systemic inflammation, a hypercoagulable state is strongly associated with the severity and mortality of COVID-19 (28). DD is not only a specific biomarker for hypercoagulability but also a biomarker for monitoring inflammation and severe infection (29). According to a recent report, along with COVID-19 progression, DD level is closely related to severity (30). Our study found that DD was significantly higher in the deceased group (*P* < 0.01).

The predictive model developed in this study has important implications for managing older patients with COVID-19. With early identification of those at high risk of mortality, clinicians can effectively allocate resources and implement timely interventions. The reliance of the model on readily available blood markers makes it practical for widespread application, potentially improving outcomes in resource-limited settings. Furthermore, the predictive accuracy of the model could support personalized treatment strategies, enabling a more tailored management of older patients with COVID-19. This might involve early initiation of antiviral therapies, immunomodulatory treatments, or other supportive care measures based on the risk profile of an individual. The combination of ML and extensive laboratory data offers a rapid and reliable technique to assist clinicians in disease differentiation.

Our study has some limitations. First, the sample size was relatively small. Second, the data were derived from a single center and were retrospective. Future studies should consider integrating this model with additional clinical and imaging data to enhance its predictive capabilities. Finally, in the present study, the patients diagnosed with tumors or severe blood system disorders or those who have received blood transfusions were excluded. However, these patients are particularly vulnerable to SARS-CoV-2 infection and prone to poor outcomes and death. Therefore, these patients should be included in future studies to confirm the results or to assess improvements to the model. Although the peak of COVID-19 has passed, the SARS-CoV-2 virus remains present, and humans continue to be intermittently infected. Thus, this study maintains its relevance and value.

## 5 Conclusion

This study identified NMR, NLR, CRP, IL6, and DD as factors associated with the risk of COVID-19-related death using LASSO regression. Assessing nine ML models, the GNB model was the best performing, demonstrating superior predictive accuracy and clinical benefit. The correlation between the actual and predicted probabilities was strong, and the online prediction tool directly contributed to improved predictions of disease progression and mortality risk in older patients with COVID-19.

## Data Availability

The original contributions presented in the study are included in the article/**Supplementary Material**. Further inquiries can be directed to the corresponding author.
