# Supplementary material for: Prediction of the risk of mortality in older patients with coronavirus disease 2019 using blood markers and machine learning
Source: Front Immunol. 2024 Nov 1;15:1445618. doi: 10.3389/fimmu.2024.1445618 (PMC11563789; doi:10.3389/fimmu.2024.1445618)
Supplement: Supplementary file 1 [file DataSheet1.pdf]

Supplement Table 1 Diagnostic efficacy of nine classifiers in the training and validation cohorts （include PCT as the 6th feature ）

Classifier	Cohorts	AUC	Cutoff	Accuracy	Sensitivity	Specificity	Positive predictive value	Negative predicitive value	F1
XGBoost	Training	1.000	0.773	0.992	0.965	1.000	1.000	0.990	0.982
	Validation	0.858	0.773	0.765	0.307	0.937	0.667	0.778	0.417
Logistic	Training	0.939	0.146	0.837	0.895	0.820	0.586	0.966	0.707
	Validation	0.905	0.146	0.838	0.950	0.801	0.632	0.976	0.755
LightGBM	Training	1.000	0.813	0.992	0.965	1.000	1.000	0.990	0.982
	Validation	0.926	0.813	0.809	0.325	0.981	0.833	0.803	0.467
RandomForest	Training	1.000	0.500	0.992	0.965	1.000	1.000	0.990	0.982
	Validation	0.893	0.500	0.794	0.600	0.862	0.611	0.860	0.604
AdaBoost	Training	1.000	0.522	0.992	0.968	1.000	1.000	0.990	0.984
	0.910	0.522	0.838	0.500	0.926	0.661	0.878	0.558	0.463
DecisionTree	Training	1.000	1.000	0.765	0.000	1.000	NaN	0.765	NaN
	Validation	0.704	1.000	0.794	0.000	1.000	NaN	0.794	NaN
GBDT	Training	1.000	0.973	0.992	0.968	1.000	1.000	0.990	0.984
	Validation	0.694	0.973	0.824	0.333	0.884	0.273	0.889	NaN
GNB	Training	0.934	0.023	0.811	0.919	0.779	0.556	0.969	0.691
	Validation	0.902	0.023	0.824	0.917	0.791	0.550	0.979	0.688
CNB	Training	0.628	0.940	0.780	0.483	0.864	0.500	0.856	0.489
	Validation	0.409	0.940	0.735	0.278	0.900	0.500	0.779	0.326

Supplement Table 2 Diagnostic efficacy of LGBM model in the testing and validation cohorts （include PCT as the 6th feature）

Cohorts	AUC	Cutoff	Accuracy	Sensitivity	Specificity	Positive predictive value	Negative predicitive value	F1

Training	1.000	0.780	0.990	0.957	1.000	1.000	0.988	0.978
Validation	0.911	0.780	0.822	0.495	0.953	0.822	0.827	0.605
Testing	0.899	0.846	0.824	0.143	1.0	1.0	0.818	0.25

Supplementary Table3 The optimized hyperparameters for the ML models

Algos	hyperparameters
XGBoost	1. objective: binary:logistic<br>2. learning_rate: None<br>3. max_depth: None<br>4. min_child_weight: None<br>5. reg_lambda: None
Logistic	1. C: 1.0<br>2. max_iter: 100<br>3. penalty: l2<br>4. tol: 0.0001
LightGBM	1. boosting_type: gbdt<br>2. learning_rate: 0.1<br>3. max_depth: -1<br>4. n_estimators: 100<br>5. num_leaves: 31
RandomForest	1. criterion: gini<br>2. max_depth: None<br>3.min_impurity_decrease: 0.0<br>4. n_estimators: 20
AdaBoost	1. learning_rate: 1.0<br>2. n_estimators: 50
DecisionTree	1. criterion: gini<br>2. max_depth: None<br>3. min_samples_leaf: 1<br>4. min_samples_split: 2
GBDT	1. learning_rate: 0.1<br>2. loss: log_loss<br>3. max_depth: 3<br>4. min_samples_leaf: 1<br>5. min_samples_split: 2<br>6. n_estimators: 100
GNB	1. priors: None<br>2. var_smoothing: 1e-09
CNB	alpha (Laplace/Lidstone): 1.0

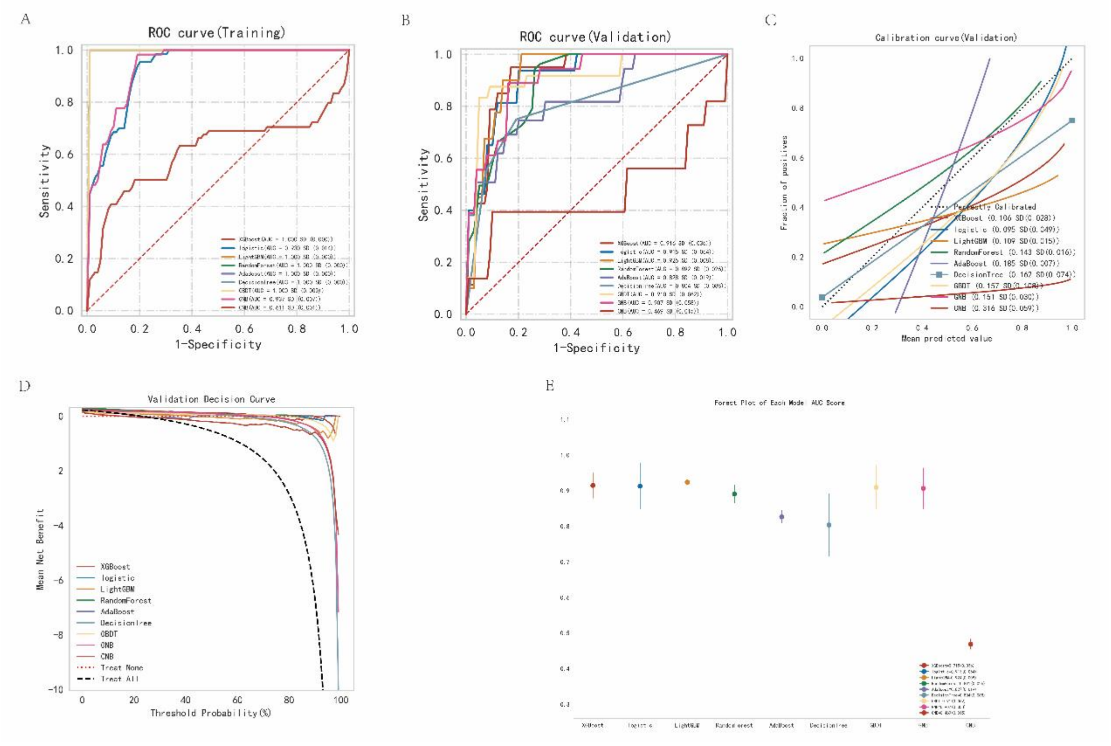

Supplement Figure1 Performance comparison between multiple models（include PCT as the 6th feature）. (A) Receiver operating characteristic (ROC) curve of training cohort; (B) ROC curve of validation cohort; (C) Calibration curve of the nine machine-learning models; (D) Decision curve of the nine machine-learning models; (E) Forest plot of each area under the curve (AUC) score.

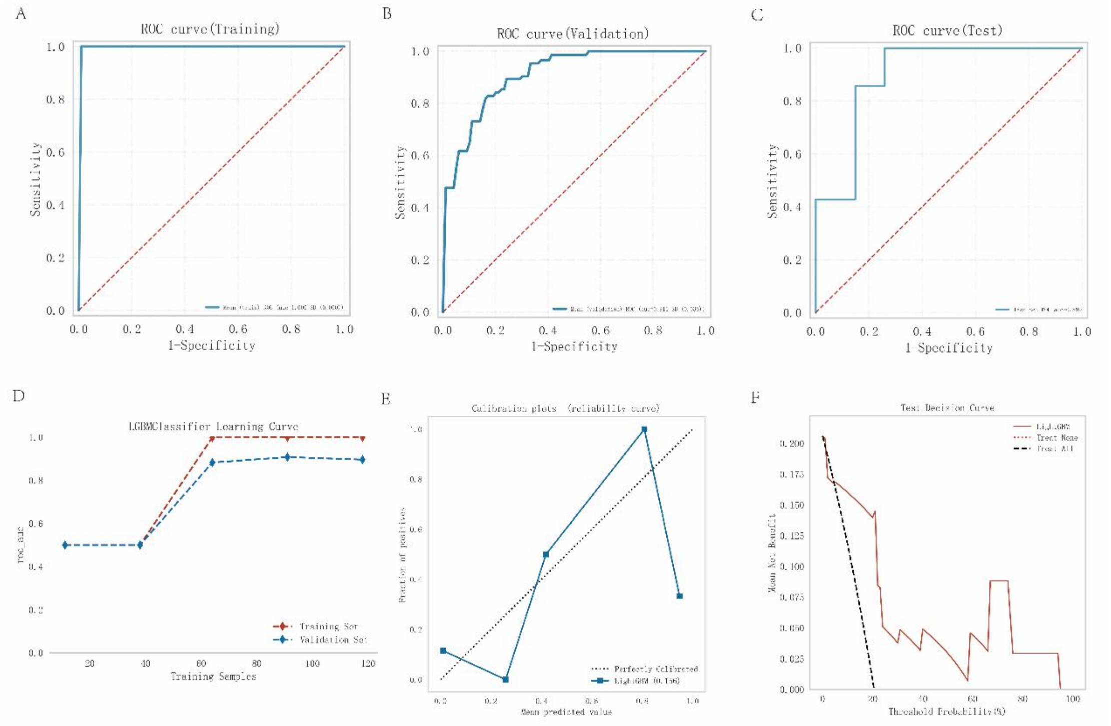

Supplement Figure2 Performance of the prediction model（include PCT as the 6th feature）. (A) Receiver operating characteristic (ROC) curve of training cohort; (B) ROC curve of validation cohort; (C) ROC curve of testing cohort; (D) AUC of validation cohort and testing cohort; (E) Calibration curve analysis; (F) Decision curve analysis.

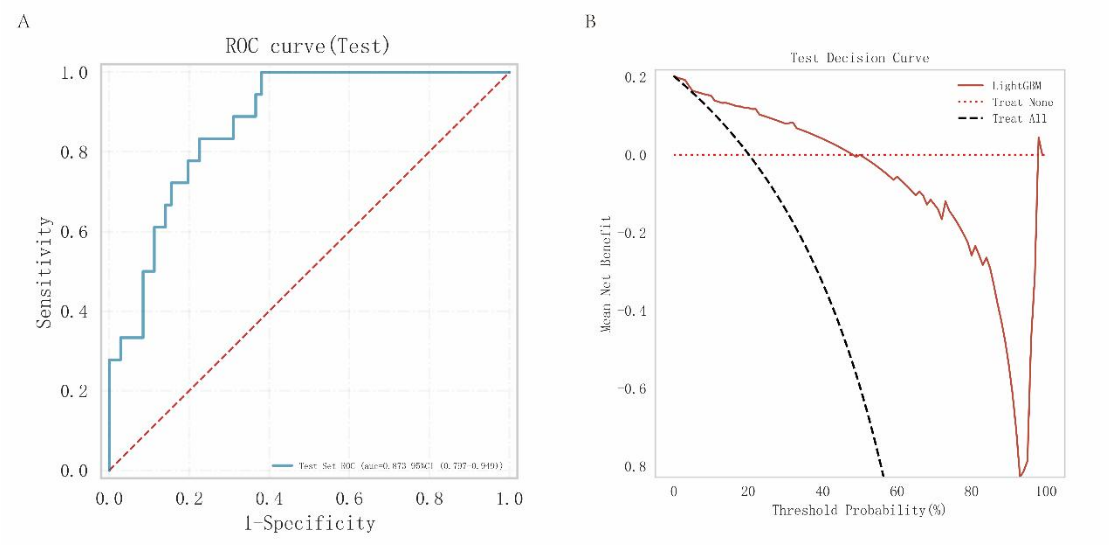

Supplement Figure3 External independent testing of LGBM regression model（include PCT as the 6th feature）. (A) Receiver operating characteristic (ROC) curve of external independent testing cohort. (B) Test decision curve of external independent testing cohort.
